# Healthcare professionals' perceived stigmatization about direct care of COVID-19 Patients: development and validation of the discrimination about COVID-19 (DisCOV-19) instrument

**DOI:** 10.4314/ahs.v23i4.11

**Published:** 2023-12

**Authors:** Abdulmuminu Isah, Chinelo Nneka Aguiyi-Ikeanyi, Chibueze Anosike Azubuike Ekwuofu, Deborah Oyine Aluh, Blessing Onyinye Ukoha-kalu, Maxwell Ogochukwu Adibe, Chinwe Victoria Ukwe, Abubakar Sadiq Abdullahi

**Affiliations:** Department of Clinical Pharmacy and Pharmacy Management, University of Nigeria, Nsukka, 410001, Enugu State Nigeria

**Keywords:** COVID-19, Discrimination, Healthcare professionals, Patient care, Questionnaire development

## Abstract

**Background:**

The novel coronavirus disease 2019 (COVID-19) is a public health concern worldwide. Healthcare professionals are among the most vulnerable groups in the fight against COVID-19 because they are directly involved in the care of at-risk persons and patients with Covid-19.

**Objectives:**

This study aimed to measure the level to which healthcare workers feel that they can be discriminated due to their involvement in the direct care of COVID-19 patients.

**Methods:**

A cross-sectional online survey was conducted among healthcare professionals in Nigeria. A nineteen-item discrimination against COVID-19 (DisCOV-19) questionnaire was developed and validated for the study. Descriptive statistics and One-Way Analysis of Variance were used for data analysis. *P*<0.05 was considered statistically significant.

**Results:**

Out of the 286 healthcare practitioners that participated in the study, 58.4% and 30.1% were pharmacists and physicians, respectively. The majority of the participants were at least “moderately concerned” about disability (60.9%), death (71.7%), unknown complications (65.1%), and risk of infecting family members and friends (83.2%) if asked to provide care for COVID-19 patients. The physicians had a significantly higher mean discrimination score compared to the pharmacists (*p*=0.041). Pharmacists had a significantly lower mean discrimination score than the nurses (*p*=0.011).

**Conclusions:**

Many of the healthcare professionals reported a certain level of concern and perceived that they could face some forms of discrimination for providing care to COVID-19 patients.

## Introduction

The novel coronavirus disease 2019 (COVID-19) is presently a public health emergency worldwide. The deadly disease was first diagnosed in December 2019 in Wuhan, China 1. On March 11, 2020, the World Health Organization (WHO) declared the COVID-19 outbreak as a pandemic because of its severity and its rapid spread to many countries around the world 2. The disease is characterized by pneumonia-like symptoms such as fever, dry cough, breathing difficulty, and fatigue 1. Individuals, especially the elderly, with co-existing chronic diseases, including diabetes and cardiovascular diseases, are at a higher risk of developing fatal complications if they are infected with COVID-19. The infection is highly contagious, hence the fear associated with the disease. It is primarily transmitted from one person to another through the oro-nasal droplets of an infected person and by direct contact 1. Nigeria, the most populous black country in Africa, announced its index case of COVID-19 in the commercial city of Lagos on February 27, 2020^3^. Since then, the disease has continued to spread through the nooks and crannies of Nigeria. Unfortunately, the number of healthcare practitioners who have tested positive to the virus continues to increase 4.

Healthcare professionals are among the most vulnerable groups in the fight against COVID-19^5^. This is because they are directly involved in the care of at-risk persons and patients with COVID-19. In disease outbreaks, healthcare professionals conduct diagnostic tests, treat, monitor, and evaluate treatment outcomes in individuals with suspected or confirmed Covid-19 disease 6.

Generally, stigma among healthcare practitioners is characterized by behaviours such as labelling, stereotyping, and separation, with overall consequential loss of status and discrimination toward patients needing medical care and attention 7. Discrimination implies bias and unjust actions directed at specific persons or groups based on perceived or real attributes, medical condition (e.g., COVID-19), financial status, gender, ethnicity, or age 8. Therefore, discrimination is summed up as the end product of stigma. Stigma among healthcare practitioners is known to negatively affect people seeking health services when they appear most vulnerable. It undermines patients' access to diagnosis, treatment, and successful health outcomes. Stigma on the part of health workers may lead to the provision of sub-standard care, physical or verbal abuse of patients, outright withdrawal of services, or transfer of patient care to less experienced colleagues 9,10. In view of COVID-19 pandemic, stigmatizing attitude among healthcare workers may be driven by shortage of personal protective equipment (PPE), highly contagious nature of the disease, unavailability of cure or vaccine, risk of contracting the infection, increased risk of exposure of family members and friends to COVID-19, and fear of complications and disabilities if infected, or even death 11–15. However, there is no available survey instrument to assess healthcare professionals' perceived discrimination towards providing direct care for COVID-19 patients. This study aimed to measure the level to which healthcare workers feel that they can be discriminated due to their involvement in the direct care of COVID-19 patients.

## Methods

### Study design

This study is a cross-sectional online questionnaire-based design, conducted on healthcare professionals in Nigeria.

### Study setting

The study was conducted at the University of Nigeria, African Health Sciences, Vol 23 Issue 4, December, 2023 Nigeria's first-degree awarding institution. The institution has many healthcare professionals that are under its employment, either as academic or clinical staff. There is a 20-bed capacity medical centre at the main campus of the university which provides primary and secondary levels of healthcare to the members of the University community. The university also has a gigantic referral teaching hospital which serves both as a specialist treatment centre, as well as a training centre for undergraduate and postgraduate health sciences students.

### Study sample

The Physicians, pharmacists, and nurses who were resident at the teaching hospital directly providing care for COVID-19 patients who agreed to be a part of this study were used for this study. The names and contacts of the staff were obtained from the administrative office. However, an opportunity was given to other healthcare professionals, such as physiotherapists and laboratory scientists, who could also have direct but less duration of contact with the patients. Healthcare professionals in all settings of practice were recruited after obtaining their informed consents to participate in the study. A three-week time-based sampling was conducted, using the WhatsApp Groups of the intended healthcare professionals in the institution.

### Development and validation of the study instrument

An extensive literature search of both print and electronic resources was conducted at the initial stage of this cross-sectional study. This was done to obtain nineteen questions that directly relate to discrimination concerns of healthcare professionals attending to infectious diseases, especially those that were difficult to treat, yet with high transmission rate. A similar method of data collection instrument development is found in a cross-sectional study on factors associated with healthcare workers' willingness to participate in disasters management in Sana'a Yemen, Southwest Asia. In that study, the study instrument was similarly developed based on previous studies and opinions of national and international experts. The draft questionnaire was pretested for validity and reliability and subsequently distributed to national and international experts to test its consistency, relatedness, representativeness, and clarity of wording. At the end, a reliable instrument with internal consistency was obtained, considering the Cronbach's alpha scores 17. All the questions had similar Likert scale responses of not at all, a little, moderately, and a lot. The instrument reliability was also determined as confirmed by acceptable Cronbach Alpha of 0.92. Content validation was done by presenting the draft questions to ten 10 experienced academic and clinical healthcare professionals at the different practise areas. They were asked to score each question for relevance, accuracy, ambiguity, and appropriateness to measure the study variable. Acceptable content validation ratios (at least 0.99) were determined using Lawshe's formula. The content-validated instrument was then presented to ten healthcare professionals of the University of Nigeria Medical Centre for face validation. The primary role was to determine the ease of understanding the questionnaire's wordings and the intended meaning of each question. The draft questionnaire was converted into a Google Form whose link was shared with the WhatsApp Groups of the various healthcare professionals. Reminders were sent to the groups every other day for the three weeks of the data collection period.

### Data management and analysis

At the end of the designated period for data collection, receipt of response was switched off in the Google account. The collated responses were downloaded in a .cvs format into Microsoft Excel (2016) for data cleaning. The final accepted data were analysed using KwikTables© and IBM Statistical Products and Services Solution (SPSS) Version-25. The four responses of the discrimination questions were scored as 0, 1, 2, and 3 for not at all, a little, moderately, and a lot, respectively. The mean of each respondent's performance was determined as a factor of 1 by summing all their responses and dividing them by the total maximum possible score. The participants were grouped into four levels of perceived discrimination using the percentile scores. Frequencies, percentages, means, and standard deviations (SD) were used to describe the participants' responses. One-Way Analysis of Variance (ANOVA) was used to compare the mean scores of the respondents, with LSD post-hoc test conducted for between-group comparison. For all analyses, p values < 0.05 were considered to be statistically significant.

### Ethics approval and consent to participate

Ethical approval for this study was obtained from the University of Nigeria Teaching Hospital Ethical Committee and the Ethical Clearance Certificate reference number is NHREC/05/01/2008B-FWA00002458-1RB00002323. All the participants were properly informed of the voluntary nature of the study, and informed consent was obtained from all of them through direct phone calls.

## Results

Table 1 shows the sociodemographic characteristics of the respondents. The number of responses obtained was 286, from a total of 405 healthcare professionals that were contacted (70.62 % response rate). Out of the 286 healthcare practitioners, 167(58.4%) and 86(30.1%) were pharmacists and physicians, respectively. More than half of the respondents, 157(54.90%), were males. The majority of the participants, 182(63.6%), were aged 30–49 years old and were public sector employees, 186(65.0%). The average years of experience in practice of the participants was 8.5 ± 6.6 years.

**Table 1:** Sociodemographic characteristics of the healthcare professionals

Characteristics	Nurses	Pharmacists	Physicians	Others	Total	*P*-value*
	(N=27)	(N=167)	(N=86)	(N=6)	(N=286)	
Age (years), n (%)						< 0.001
< 30	4 (14.8)	72 (43.1)	17 (19.8)	0 (0.0)	93 (32.5)	
30 – 39	14 (51.9)	65 (38.9)	39 (45.3)	5 (83.3)	123 (43.0)	
40 – 49	9 (33.3)	19 (11.4)	30 (34.9)	1 (16.7)	59 (20.6)	
50 – 59	0 (0.0)	9 (5.4)	0 (0.0)	0 (0.0)	9 (3.1)	
60 – 69	0 (0.0)	1 (0.6)	0 (0.0)	0 (0.0)	1 (0.3)	
≥70	0 (0.0)	1 (0.6)	0 (0.0)	0 (0.0)	1 (0.3)	
Gender, n (%)						< 0.001
Female	25 (92.6)	70 (41.9)	29 (33.7)	5 (83.3)	129 (45.1)	
Male	2 (7.4)	97 (58.1)	57 (66.3)	1 (16.7)	157 (54.9)	
Practice Sector, n (%)						< 0.001
Private	3 (11.1)	90 (53.9)	4 (4.7)	3 (50.0)	100 (35.0)	
Public	24 (88.9)	77 (46.1)	82 (95.3)	3 (50.0)	186 (65.0)	
Post-graduation Experience (years), mean (SD)	8 (3.5)	8.1 (8.2)	9.5 (4.2)	8 (1.2)	8.5 (6.6)	0.417
Public Health Experience, n (%)						< 0.001
No	1 (3.7)	35 (21.0)	4 (4.7)	0 (0.0)	40 (14.0)	
Yes	26 (96.3)	132 (79.0)	82 (95.3)	6 (100.0)	246 (86.0)	

* *P*-values are from a Chi-square test for categorical variables and ANOVA test for continuous

Table 2 contains data on perceived discrimination by healthcare professionals towards caring for patients with COVID-19. The results demonstrate that the majority of the participants were at least “moderately concerned” about disability (60.9%), death (71.7%), unknown complications (65.1%), and risk of infecting family members and friends (83.2%), if asked to care for COVID-19 patients. However, less than 40% of the practitioners were “moderately concerned” or “a lot concerned” about discrimination by religious groups (33.9%), negative public perception (36.7%), and access to educational institutions (38.5%) after COVID-19 pandemic.

**Table 2:** Perceived discrimination of healthcare professionals about caring for COVID-19 patients (N=286)

Concerns of the Participants	Not at All	A Little	Moderately	A Lot

	Frequency (Percentage)			
1. Being treated differently by family and friends	59(20.6)	90(31.5)	79(27.6)	58(20.3)
2. Initiating or maintaining an intimate relationship	65(22.7)	86(30.1)	85(29.7)	50(17.5)
3. Being discriminated against by neighbours	74(25.9)	84(29.4)	76(26.6)	52(18.2)
4. Being treated in a negative way by colleagues	75(26.2)	84(29.4)	74(25.9)	53(18.5)
5. Finding or keeping a job afterward	100(35)	60(21.0)	65(22.7)	61(21.3)
6. Being discriminated by religious group	119(41.6)	70(24.5)	62(21.7)	35(12.2)
7. Mental health issues afterward	92(32.2)	83(29.0)	53(18.5)	58(20.3)
8. Accessing public services such as healthcare, utility, etc.	88(30.8)	76(26.6)	67(23.4)	55(19.2)
9. Being perceived in a negative way by the general public	78(27.3)	103(36.0)	48(16.8)	57(19.9)
10. Death from the infection	23(8.0)	58(20.3)	74(25.9)	131(45.8)
11. Disability from the disease	46(16.1)	66(23.1)	64(22.4)	110(38.5)
12. Being infected with the disease	22(7.7)	59(20.6)	63(22.0)	142(49.7)
13. Infecting family and friends if infected	10(3.5)	38(13.3)	59(20.6)	179(62.6)
14. There may be no cure if I am infected	43(15.0)	70(24.5)	79(27.6)	94(32.9)
15. The long-term discrimination afterward	76(26.6)	81(28.3)	77(26.9)	52(18.2)
16. Unknown complications from the disease	31(10.8)	69(24.1)	76(26.6)	110(38.5)
17. Being attacked by the patients	47(16.4)	90(31.5)	82(28.7)	67(23.4)
18. Accessing services from educational institutions afterward	104(36.4)	72(25.2)	74(25.9)	36(12.6)
19. Life may not be the same for me afterward	63(22)	92(32.2)	80(28.0)	51(17.8)

Table 3 shows the classification of healthcare professionals based on the discrimination level. More than half of the physicians demonstrated moderate perceived discrimination (37.2%) or a lot of perceived discrimination (20.9%). Approximately 16.8% and 28.1% of the pharmacists had moderate discrimination or a lot of discrimination towards caring for COVID-19 patients. On the other hand, more than half of the nurses (55.5%) had moderate perceived discrimination. About a quarter (25.9%) feared a lot of discrimination. Nonetheless, about 33% of pharmacists, the highest compared to other health practitioners, had no perceived discrimination caring for COVID-19 patients.

**Table 3:** Classification of respondents by discrimination level

Health Profession	Perceived Discrimination Level	Frequency	Percentage
Physicians	No Perceived Discrimination	16	18.6
	A Little Perceived Discrimination	20	23.3
	Moderately Perceived Discrimination	32	37.2
	A Lot of Perceived Discrimination	18	20.9
	Total	86	100.0
Pharmacists	No Perceived Discrimination	55	32.9
	A Little Perceived Discrimination	37	22.2
	Moderately Perceived Discrimination	28	16.8
	A Lot of Perceived Discrimination	47	28.1
	Total	167	100.0
Nurses	No Perceived Discrimination	1	3.7
	A Little Perceived Discrimination	4	14.8
	Moderately Perceived Discrimination	15	55.6
	A Lot of Perceived Discrimination	7	25.9
	Total	27	100.0
Others	No Perceived Discrimination	1	16.7
	A Little Perceived Discrimination	3	50.0
	Moderately Perceived Discrimination	2	33.3
	Total	6	100.0

Table 4 shows the multiple comparisons of the participants' mean discrimination score by the health profession. The physicians reported a significantly higher mean discrimination score compared to the pharmacists (p = 0.041). Similarly, pharmacists had a significantly lower mean discrimination score relative to the nurses (p = 0.011)

**Table 4:** Multiple comparison of the participants' mean discrimination scores according to their profession

(I) Health Profession	(J) Health Profession	Mean Difference (I-J)	Std. Error	Sig.	95% Confidence Interval	
					Lower Bound	Upper Bound
Physicians	Pharmacists	0.062*	0.030	0.041	0.003	0.121
	Others	0.080	0.096	0.402	-0.108	0.268
	Nurses	-0.058	0.050	0.242	-0.157	0.040
Pharmacists	Physicians	-0.062*	0.030	0.041	-0.120	-0.003
	Others	0.019	0.094	0.844	-0.167	0.204
	Nurses	-0.120*	0.047		-0.213	-0.028
Others	Physicians	-0.080	0.096	0.402	-0.268	0.108
	Pharmacists	-0.019	0.094	0.844	-0.204	0.167
	Nurses	-0.139	0.102	0.175	-0.340	0.062
Nurses	Physicians	0.058	0.050	0.242	-0.040	0.157
	Pharmacists	0.120*	0.045		0.028	0.213
	Others	0.139	0.102	0.175	-0.062	0.340

* The mean difference is significant at the 0.05 level.

## Discussion

Majority of the respondents in this study were aged between 30 – 39 years. The eagerness to respond to the study is expected, as previous studies have shown a directly proportional relationship between age and prejudice toward people living with high-risk infectious diseases 18. This age predisposition suggests that older people may not readily enlist in the direct care for patients with high-risk infectious diseases, compared to the younger professionals. An apparent variance was observed between gender distribution and the health professionals' careers; a larger proportion of females were nurses and a larger number of males were physicians and pharmacists. This is likely due to perceived career roles, the influence of family, peers, and general societal perceptions of gender preferences for specific professions 19.

The study participants' greatest concerns were the possibility of being infected with the disease, infecting their family and friends if they became infected, and dying from the infection if they become infected. In Nigeria, the results of a cross-sectional study on the differences between health workers and the general population in risk perception, behaviours, and psychological distress related to COVID-19 revealed that healthcare workers generally worry about the rapid spread of the virus and the possibility of being isolated 20. In Japan, recent study also revealed that the healthcare professionals showed about 2.5 times higher odds of perceiving themselves at risk of infection compared to the general population 19. A similar study by Lai et al., in a recent cross-sectional, survey-based, and region-stratified study on the factors associated with mental health outcomes among healthcare workers in China identified issues such as psychological distress, anxiety, depression, and insomnia. The study also argued that the intensified perception of personal danger among healthcare workers might be due to the potentially fatal, human-to-human transmissibility, and associated high morbidity of COVID-19^22^. These studies suggest that the willingness of healthcare workers to provide adequate care, void of discrimination, to patients is directly linked to their perceived level of protection 23. Thus, protecting health care workers is an essential component of public health measures for addressing the COVID-19 epidemic. This could also be facilitated by implementing specialized interventions to promote mental well-being in health care workers exposed to COVID-19^23^,^24^.

The majority of the healthcare workers were moderately concerned about their lives never remaining the same, and the accessibility to the medications for treating COVID-19. Respondents were also concerned a little about being perceived negatively by the public, being attacked by patients, and being treated differently by family and friends. A broad review on all types of articles on mental health problems faced by healthcare workers due to the COVID-19 pandemic conducted between January 2020-Apri 2020 revealed that personal safety, concerns for their families, and concerns for patient mortality, exhaustion from work, lack of personal protective equipment, the safety of colleagues and the lack of treatment for COVID-19 were perceived as factors of concern which consequently resulted into induced stress in all healthcare workers^25^. This is further buttressed by the work of Mohanty et al. who outlined other concerns to be the probability of a healthcare worker being taken as an object of undeserved attack, false accusations and law suits and dangerous hazards 26. A practical approach towards combatting these concerns would require the intensification and communication of research findings, enhancement of isolation centres, optimization of shift duties, and general protection and promotion of healthcare workers' personality.

The significance of public and social supports on healthcare professionals' perceived discrimination could not be overemphasized. A one-month cross-sectional observational study on the effects of social support on sleep quality of medical staff treating patients with coronavirus disease in China between January and February 2020 showed that social support contributed to improving self-efficacy, and a sense of professional achievement which ultimately resulted in healthcare workers suffering less from loneliness, increased optimism and improved coping mechanisms when under stress 22. Therefore, the public needs to understand that they also have a role in fostering the optimal performance of healthcare workers. This objective can be achieved through mass media, awareness campaigns, and community enlightenment. Healthcare workers in the present study were least concerned about accessing services from educational institutes, keeping and finding a job post-pandemic. These data should thus serve as a pointer for policy makers and healthcare administrators to be keen about not investing scare resources in areas, not of significant concerns to healthcare workers.

Analysis of the results of this study shows an ascending order of perceived discrimination among pharmacists, physicians, and nurses. This correlates with the findings of a web-based cross-sectional study on anxiety and depression in health workers and the general population during the COVID-19 epidemic in Iran. The study found that anxiety and depression were significantly more prevalent in physicians and nurses compared with other occupations 27. Similarly, a cross-sectional observational study on the psychological impact and coping strategies of frontline medical staff during the outbreak of coronavirus disease 2019 in Hubei, China, between January and March 2020 reported that nurses felt more anxious and nervous compared to other professionals 25. This increased anxiety and perceived discrimination among physicians and nurses may be due to the longer contact time they spend with COVID-19 patients. Simione et al. demonstrated a correlation between perceived direct exposure to COVID-19 and disease-related concern or anxiety. He also described the relationship between past experiences and disease-related concerns or anxiety 28. These assertions find greater prominence following the fact that during the 2014 Ebola outbreak, most healthcare workers who died in West Africa were physicians and nurses 29. These bitter incidences suggest the rationale behind the more significant concern, anxiety, and discrimination among physicians and nurses in this study. A useful measure to curb this menace would be to organize more profession-specific public health campaigns that would address the concerns of healthcare professionals about COVID-19.

## Limitations of this study

The study had limited scope as all its respondents were from one institution, limiting the study's generalizations to other settings with different healthcare systems. However, the questions in the instrument are applicable to all settings of healthcare practice. In addition, this study was a snapshot, thus lacks longitudinal follow-up. Due to the increasing prevalence and impact of COVID-19, the perceived discrimination of healthcare workers in Nigeria could become more severe. Furthermore, this study was unable to distinguish pre-existing and emergent perceived discrimination status among the study population. Lastly, considering the current workload of frontline workers, many of the eligible respondents may not have been able to participate in this study, even if they were willing to do same.

## Conclusion

This study developed and validated DisCoV-19, a nineteen-item novel instrument to evaluate the perceived discrimination of healthcare professionals towards caring for patients with COVID-19. Many of the healthcare professionals that were studied in this research reported a certain level of concern and perceived that they could face some forms of discrimination for providing care to COVID-19 patients. Their main concerns were the fear of death and unknown disability from the disease, while many were unconcerned about the possibility of a mental health problem or discriminatory treatment from peers in the healthcare community.

## Recommendation

There should be intensification and communication of research findings on Covid-19 related matters so as to enlighten and educate the health workers and general public. There is need for enhancement of healthcare workers' personality, and organization of profession-specific public health campaigns that would address healthcare professionals' specific concerns on COVID-19. Public sensitization programs should also enlighten the public on the significance of offering social support and recognition to healthcare workers. Only collaborative efforts can yield a successful fight against COVID-19. Nevertheless, this study is a timely one that has shown the major concerns of healthcare workers that demand an immediate and proactive approach.

## Data Availability

The data related to the study are attached as a supplementary material
